# Social support as coping mechanism mediates the relationship between eco-anxiety and pro-environmental behavior

**DOI:** 10.1371/journal.pone.0326931

**Published:** 2025-07-10

**Authors:** Veera Nieminen, Sonja Palmo, Katriina Hyvönen, Jane-Veera Paakkolanvaara, Kaisa J. Raatikainen, Joona Muotka

**Affiliations:** 1 University of Eastern Finland, Department of Psychology, Kuopio, Finland; 2 Finnish Institute for Health and Welfare (THL), Environmental Health Unit/Environmental Epidemiology, Kuopio, Finland; 3 University of Oulu, Faculty of Education and Psychology, Oulu, Finland; 4 Institute of Rehabilitation, JAMK University of Applied Sciences,; 5 Finnish Environment Institute (Syke), Societal Change Unit, Jyväskylä, Finland; 6 University of Jyvaskyla, Department of Biological and Environmental Science, School of Resource Wisdom, Jyväskylä, Finland; Yunnan University of Finance and Economics, CHINA

## Abstract

We studied social support as a coping strategy for eco-anxiety. Furthermore, we investigated the moderating or mediating role of social support in the relationship between eco-anxiety and pro-environmental behavior (PEB). The participants were Finnish adults aged 19–42. Most of the participants were women (76.2%), lived in an urban environment (82.7%), and were students (47.4%) or employees (42.8%). Data collected with an online survey was analyzed using correlation analysis, hierarchical regression analysis, and a simple mediation model. We found that experiencing eco-anxiety was positively related to PEB, and that the participants with higher social support had higher levels of eco-anxiety. In addition, using social support as a coping strategy was connected to a higher level of PEB. We found that social support mediates the positive connection between eco-anxiety and PEB, but no moderating effect was found. Our results imply that social support can be an important coping strategy for adults coping with eco-anxiety, as well as promoting PEB, offering an interesting insight for future interventions.

## Introduction

We live in the era of environmental crises, of which the most pressing – or at least most discussed – are global climate change and biodiversity loss. These global environmental issues affect our lives in many ways, not just in the future but right here and now. Recently, there has been growing interest in studying how climate change and other environmental issues influence our well-being, and research has begun to better understand the distress and anxiety arising from climate change [1–8], the most discussed environmental issue. One of the most topical forms of psychological effects of environmental issues is eco-anxiety.

Another growing focus in the field of psychology has been the study of pro-environmental behavior (PEB). In the present study, we examine the relationship between eco-anxiety and PEB. The research on the subject has been contradictory. For example, Ogunbode and colleagues [1], and Heeren and others [9] found climate anxiety to be positively associated with PEB. On the contrary, some studies have found a negative association [10] whereas some did not find any connection between PEB and climate anxiety [11]. We speculate that part of this inconsistency in the relationship between eco-anxiety and PEB could be explained by exploring the potential underlying factors that influence the relationship between the two concepts. Therefore, we suggest it is important to gain insights into the factors that strengthen the link between eco-anxiety and pro-environmental behavior and to examine factors that reduce PEB. The objective of this study is to contribute to current understanding of factors that strengthen the link between eco-anxiety and PEB, specifically focusing on the role of social support as a coping strategy in the relationship between eco-anxiety and PEB. Understanding eco-anxiety and the coping strategies associated with it can help both to address these emotions and to reinforce responsible environmental behavior.

### Environmental issues and eco-anxiety

Environmental issues, of which climate change is the most studied, have both direct and indirect effects on mental health [7,12]. Direct effects refer, for example, to the acute effects of extreme weather events and changes in the environment that have immediate consequences for mental health (e.g., stress, anxiety, depression, PTSD) [7,12]. In turn, the indirect consequences result from uncertainty and concern about future risks and from people’s increased awareness of climate change, rather than from people’s own experiences of climate change [7,12]. Even though eco-anxiety may result directly from environmental issues, experiencing eco-anxiety does not require direct personal experience of climate change or other environmental issues in one’s own life [6,13]. Increasing and deepening understanding of climate change, according to Fritze and colleagues [7], is causing significant harm to people’s social, emotional, and mental well-being. For example, while being worried is a natural response to environmental threats such as climate change, an inability to cope with emotions caused by environmental crises may lead to lowered psychological well-being [1,3,6,11,12].

One of the most discussed mental-health effects of climate change and other environmental problems is eco-anxiety. Eco-anxiety has been defined as an “emotional response to the threat posed by the climate and biodiversity crisis” [13]. Eco-anxiety has been a growing research interest in terms of its sub-concept, climate anxiety. Despite the fact that most studies mentioned in this article have focused on climate anxiety, we chose to use the broader term, eco-anxiety, as – in line with the definition by Hickman [13] – many people experience eco-anxiety in relation to other environmental problems in addition to the climate crisis.

Eco-anxiety can be seen as various difficult emotions related to the state of the environment and the awareness of it [6]. Emotions triggered by climate change can include sadness, depression, fear, numbness, helplessness, hopelessness, anger, and frustration [7,14,15]. Eco-anxiety can be used as a wider term that illustrates the different levels of anxiety caused by environmental issues [6,16]. In this paper, we use eco-anxiety as an umbrella term that consists of a number of negative emotions stemming from environmental issues.

### The relationship between eco-anxiety and pro-environmental behavior

The term pro-environmental behavior (PEB) refers to actions taken by individuals that aim either at diminishing environmental harm or restoring the natural environment [17]. PEB can take a number of different forms [17,18]: it may be private (for example, limiting energy consumption, avoiding waste, or recycling) or public (for example, environmental activism, public transport) [19,20] or, as Whitmarsh [18] states, individual-level, community-level or political-level action. Characteristic of these pro-environmental behaviors is that they are intentional and freely selected by an individual [19], and even though societal structures affect pro-environmental behaviors, engaging in PEB is in the end a personal choice made by an individual [20].

The relationship between PEB and eco-anxiety has been contradictory. For example, Ogunbode and colleagues [1] found that climate-anxiety was positively related to PEB. Likewise another study [21] climate-anxiety explained 32% of the variation of PEB. Roeser [22] also sees emotions as a key factor in changing an individual’s behavior towards a more environmentally-friendly direction. Environmental emotions challenge individuals to weigh their morals and motivate them to act accordingly Roeser [22]. Emotions – as well as eco-anxiety, which includes a wide range of environmental emotions – can therefore be seen as factors that influence an individual’s motivation to act. For example, worry, which is one of the emotions linked to eco-anxiety, has also been seen as a motivator, driving individuals to seek solutions and take action [14]. In contradiction, Stanley and colleagues [10] discovered a negative connection between eco-anxiety and collective PEB and found no connection to individual PEB. Clayton and Karazia [11] did not find a connection between climate anxiety and PEB.

### Social support as a coping strategy

In the context of eco-anxiety, gaining an understanding of coping strategies could offer insights into the contradictory findings regarding the relationship between eco-anxiety and PEB. Doherty and Clayton [12] suggest that psychological adaptation to climate change and other environmental issues can be characterized in the same way as adaptation to other problems. As mentioned earlier, anxiety can serve as a motivator to seek solutions and take action. However, as Ojala and colleagues [14] state, the failure to cope with eco-anxiety could also result in paralysis.

Coping strategies are an individual’s means of adapting to difficult situations that the individual perceives as beyond their resources [23]. Coping strategies can be divided into problem-focused (solving the situation) and emotion-focused (adapting to the situation) strategies [23]. Social support as a coping strategy follows this division: on the one hand, the individual may seek advice, information, and concrete help from others (problem-focused social support). On the other hand, they may seek understanding and support for dealing with emotions and moral issues (emotion-focused social support) [24]. Accordingly, Bradley and colleagues [25] have divided social support into seeking advice and seeking comfort. In addition, previous research suggests that an individual’s perception of social support availability may influence psychological well-being and adjustment to stress more than the actual amount of received social support [26,27].

Seeking social support is a coping strategy [23] related to better well-being: social support is thought to be a protective factor against stress [28] as well as a mediating factor between a stressor and the resulting distress [29]. Barrera [29] suggests that in the absence of social support, the link between stressor and distress is strengthened. Clayton [3] states that problem-focused coping is associated with better well-being since it targets the underlying problem and aims to change it. However, in the context of climate change and other environmental problems individuals have very limited means to make a difference. If the problems are not solvable, problem-focused coping might lead to greater distress, and according to Pihkala [6] even burnout. Thus, emotion-focused coping is also necessary.

In the context of environmental issues, Reser and others [30] suggest that ideal coping strategies aim at a) corrective action and b) adaptation to the situation. Accordingly, optimal coping strategies have been suggested to include emotional regulation as well as problem solving and focus on prosocial solutions [12]. Social support appears to offer both elements. Indeed, social support has been associated with both improved psychological well-being and PEB in a study by Bradley and colleagues [25], where social support moderated the relationship between the experience of climate change and risk perception as well as risk perception and distress. In addition, perceived social support has been associated with better adjustment to stress [27] and lower depressive symptoms [26]. Thus, we conclude that higher levels of social support could be associated with lower levels of eco-anxiety and higher levels of PEB.

### Aims and purpose of the present research

Our study aims to examine the relationship between eco-anxiety and pro-environmental behavior (PEB) among environmentally aware adults aged 19–42. In addition, we investigate whether social support as a coping strategy has a mediating or moderating role in the connections between eco-anxiety and PEB. Studying the role of social support in coping with eco-anxiety is crucial for understanding PEB on an individual level and for recognizing factors related to better well-being.

The research questions:

1. Is eco-anxiety related to pro-environmental behavior (PEB)?
2. Does social support mediate or moderate the relationship between eco-anxiety and PEB?

Based on previous research [1], we hypothesize that eco-anxiety is connected to PEB (H1). However, we cannot generate a hypothesis on the explanatory power of eco-anxiety on the variation of PEB, as the current research on the topic is inconsistent, [1,10,11,21]. Social support has previously been suggested to be associated with better psychological well-being [25,26] and to protect against stress [27,28]. Since perceived social support has been found beneficial for adjusting to stress [27] we examined social support from that point of view. Furthermore, social support has also been found to be a factor associated with engaging in PEB [25]. We thus hypothesize that social support is associated with lower levels of eco-anxiety and higher levels of PEB (H2) [25]. The mediating or moderating role of social support on the association between eco-anxiety and PEB has not been previously investigated, and therefore we did not generate research hypotheses regarding social support as a moderator or mediator.

## Materials and methods

### Participants and questionnaire

The data was collected with an online questionnaire, which included questions related to emotions about environmental issues, emotions about nature, self-reported environmental actions, and background information [31]. The participants were Finnish adults aged 19–42 (M = 28, SD = 5.9). Majority of the participants were women (76.4%, n = 281), with 21.4% identifying as male (n = 79). Due to the small category size (2.2%, n = 8), the participants identifying as other were excluded from the analyses. Participants of the study were proportionately high in environmental awareness based on their background factors, with 26.9% of the participants having had worked with environmental issues and 57.6% having studied in the field related to environmental issues. The questionnaire included both multiple-choice and open-ended questions, of which the former were conducted based on earlier literature [32], and it was answered anonymously. The participants provided their written informed consent to participate in this study. The questionnaire was sent to all 34 Finnish universities and universities of applied sciences, the 12 largest labor unions in terms of membership, to the youth or student sections of three Finnish trade unions, and randomly selected upper secondary schools and vocational schools from the listing of educational institutions on a website maintained by the Ministry of Education and Culture. The upper secondary schools were divided into clusters according to their location, and 10% of the upper secondary schools were selected from each cluster using a cluster sampling method. This resulted in a total of 34 selected upper secondary schools. Ten vocational schools were randomly selected from the entire country. The survey was conducted between May 24th and June 10th, 2018, and it resulted in a total of 401 responses [31]. The participants selected were those who had answered all the compulsory questions. The total number of participants was 368. The final sample was 358 after removing background categories with small sample sizes or unspecified responses. Detailed information on background factors is summarized in Table 1 below.

**Table 1:** Background factors of the participants.

Baseline characteristic	*n*	*%*
Gender		
Women/ men/ other^a^	281/ 79/ 8	76.4/ 21.4/ 2.2
Place of residence		
Urban	305	82.7
Rural	37	10.0
Village	26	7.0
Education level		
Comprehensive or secondary education	93	25.3
University degree	250	67.9
Doctoral studies	22	6.0
Other^a^	3	0.8
Main activity		
Employed	158	42.8
Entrepreneur	5	1.4
Unemployed	14	3.8
Student	175	47.4
Other^a^	16	4.3
Number of children		
0/ 1–2/ ≥ 3	315/ 46/ 7	85.4/ 12.5/ 1.9
Monthly net income		
< 1000e	148	40.1
1000-3000e	189	51.2
> 3000e	16	4.3
Prefers not to say^a^	15	4.1

*Note.* ^a^ category removed from further analyses

### Methods and variables

**Eco-anxiety** was examined using a sum variable of environmental emotions. Environmental emotions were inquired by the question “When you think about environmental issues, how strongly do you feel the following emotions?” The question was answered on a six-point Likert scale (1 = not at all and 6 = very much). The participants were asked to rate how strongly they felt the following emotions when thinking about environmental issues: worry, sadness, hope, despair, guilt, helplessness, joy, anger, anxiety, happiness, fear, indifference, melancholy, depression, insecurity, and restlessness. To construct the sum variable of eco-anxiety, we used factor analysis compiling the items of different environmental emotions: worry, sadness, despair, restlessness, guilt, helplessness, anger, anxiety, fear, melancholy, depression, and insecurity. The items hope, joy, happiness, indifference and restlessness were excluded from the sum variable. The mean of eco-anxiety was 3.4 (SD = 1.0). The reliability of the variable can be considered good/excellent (α = 0.92) [33].

**Pro-environmental behavior (PEB)** was measured by a multiple-choice question “Which of the following have you done or chosen to do partly or completely for environmental reasons? You can select several options.” The answer options included 15 statements related to PEB, for example: “I have stopped driving my private car”, “I do not travel by plane” and “I have a vegan diet”. In addition, the multiple-choice question included the response option “I have not done any of the mentioned above”. The participants who chose this option scored 0 for the PEB variable. The variable for PEB was constructed from the environmental actions selected by the participants and the variable could have a value between 0 and 15. The higher the value, the more pro-environmental behavior the participant engaged in. The mean of the PEB variable was 6.9 (SD = 2.4). Reliability was considered poor (α = 0.57) [33], possibly due to the heterogeneity of the statements indicating different environmental actions. We decided, however, to include all the dimensions of PEB, although diverse, as they conceptually capture the essence of PEB, and as similar items have been used in previous research (see, e.g., Ogunbode et al. [1]).

**Social support** was examined using a single-item measure of whether the participant perceived they had availability to social support specific to environmental emotions. The variable was constructed from the question “Do you know someone with whom you can discuss the feelings you have about environmental issues?”. There were three response options: 1 = yes, 2 = no, and 3 = I don’t want or don’t feel the need to. Due to the small number of participants in the categories, “no” and “I don’t want or don’t feel the need to “, the response options for the social support question were recoded as two categories, as a dichotomous variable, where 1 = yes (n = 291) and 0 = no/ do not want or do not feel the need (n = 77).

**The background variables** included in the analyses were gender (1 = women, 2 = men), education level, monthly net income, number of children, main activity, age, and place of residence. We included in the analyses those background variables that correlated with the variable describing the level of PEB. For some of the variables we had to remove categories due to their small size or because of unspecified responses. The background variables and removed categories are presented in Table 1.

### Data analysis

We examined the connections between eco-anxiety and social support using Pearson and Spearman correlation coefficient tests and hierarchical regression analysis. A sum variable of eco-anxiety was constructed using factor analysis. Analyses were performed using IBM SPSS Statistics 26 and Mplus version 8.4 statistical software. We controlled for background variables (gender, place of residence, and education level) that were significantly correlated to PEB in further analyses.

### Factor analysis

To explore whether we could find a factor that theoretically corresponds to the concept of eco-anxiety, we used an exploratory factor analysis (EFA) including all measured emotions related to environmental issues. EFA was conducted using Varimax rotation and Maximum Likelihood estimation method. We were interested in identifying the most correlated emotions, including only variables with a communality above.3. We included factors with an Eigenvalue above 1, which best explained the variance of the variables. We also looked at the loadings of the variables on the factors and considered the meaning of the items when creating a mean-sum variable of eco-anxiety.

### Hierarchical regression analysis

We used hierarchical regression analysis to examine the connections between eco-anxiety and PEB. All continuous variables in the model (eco-anxiety, PEB) were standardized. First, we examined how eco-anxiety explained the level of PEB after controlling for background variables. All the background variables correlated with PEB were added to the first step of the analysis. The summary variable for eco-anxiety was added in the second step of the analysis. In addition, analyses were conducted to examine the explanatory role of eco-anxiety on PEB by adding to the model the social support variable as well as an interaction term measuring its moderating effect in addition to the background variables. The social support variable was added in the second step of the analysis, eco-anxiety in the third step and the interaction term in the fourth step.

### Mediating effects

A simple mediation model was used to examine the mediating effect of social support between eco-anxiety and PEB. The mediating effect describes how the independent variable affects the dependent variable through one or more mediating variables [34] We used eco-anxiety as the independent variable, the level of PEB as the dependent variable, and social support as the mediating variable. Social support was recoded as a dichotomous variable, and therefore the mediating effect of social support on the connection between eco-anxiety and PEB was examined using Mplus version 8.4, a software tool for examining the mediating effects of dichotomous variables. The analyses used a 95% bias-corrected bootstrap confidence interval to test the statistical significance of the indirect effect. The number of bootstrap samples was 5000. The effect from independent to mediator was performed using a probit link function. A weighted Least Square Mean and Variance Adjusted (WLSMV) estimator was used in the analyses.

## Results

### Descriptive results

#### Results of factor analysis.

**Fig 1:**
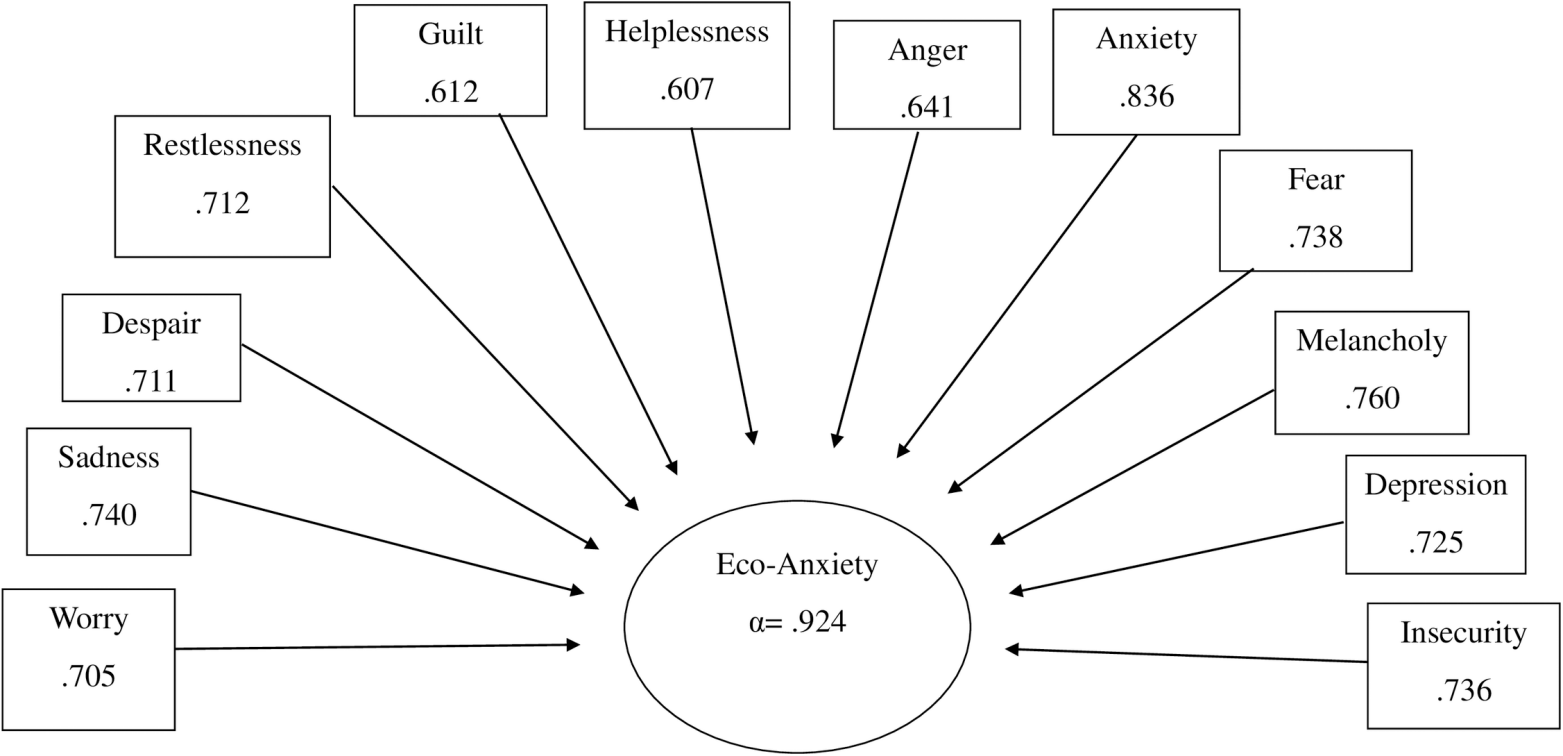
The loading of environmental emotions on a factor of the sum variable of eco-anxiety.

Variables indifference, hope, and restlessness were removed because their communalities were below.3. Two factors emerged from the analysis. The first factor theoretically corresponded to the concept of eco-anxiety (Eigenvalue 6.58, explained 47% of the variance), including all other emotions except for joy and happiness, which loaded on the second factor. As the first factor captured the essence of eco-anxiety, the second factor was excluded from further analysis. We constructed a summary variable from the variables loaded on the first factor (Fig 1).

#### Results of correlation analysis.

Eco-anxiety was connected with a higher level of PEB. Thus, correlation analyses supported our hypothesis of a connection between eco-anxiety and PEB (H1). Social support was connected with both higher levels of PEB and higher eco-anxiety. Thus, our hypothesis regarding social support being connected to higher levels of PEB was supported, whereas our hypothesis of social support being connected to lower levels of eco-anxiety was not fulfilled (H2). As for the background variables, gender, residence, and education level were connected with the level of PEB (Table 2). The correlation analyses showed that women reported higher PEB than men. Living in an urban area was connected with a higher level of PEB while living in a rural area was connected with a lower level of PEB. The participants with a primary or secondary education were less likely to have been engaged in PEB. In contrast, doctoral studies were connected with a higher level of PEB. Monthly net income, number of children, main activity at the time of the survey, and age were not correlated with PEB. Women, urban dwellers, and entrepreneurs were more likely to experience eco-anxiety. Women received more social support than men and the participants with comprehensive or secondary education received less social support than the participants with other educational backgrounds.

**Table 2:** Correlations.

Variable	1	2	3	4	5	6	7	8	9	10	11	12	13	14	15	16	17
1. PEB^a^	−																
2. Eco-anxiety^a^	.389**	−															
3. Social support^**b**^	.319**	.218**	−														
4. Gender^b^	−.231**	−.283**	−.170**	−													
5. Age^a^	0.038	0.045	0	−0.03	−												
6. No. of children^a^	−0.04	−0.053	0.024	−0.032	.483**	−											
7. Urban env.^**b**^	.158**	.103*	0.085	−0.038	0.015	−.174**	−										
8. Rural env.^**b**^	−.118*	−0.07	−0.1	0.042	−0.02	0.097	−.736**	−									
9. Village env.^**b**^	−0.093	−0.066	−0.015	0.008	0.004	.141**	−.607**	−0.092	−								
10. Compr. or second.^**b**^	−.111*	−0.01	−.145**	.135*	−.347**	−0.116	−.159**	0.102	.115*	−							
11. University degree^**b**^	0.037	0.027	0.083	−0.1	.215**	0.064	.139**	−0.072	−.120*	−.862**	−						
12. Doctoral studies^**b**^	.131*	−0.034	.103*	−0.051	.215**	0.088	0.021	−0.045	0.022	−.148**	−.373**	−					
13. Employer^**b**^	0.003	−0	0.051	−0.04	.478**	.242**	−0.02	−0.01	0.04	−.268**	.144**	.207**	−				
14. Student^**b**^	−0.05	−0.05	−0.07	0.063	−.490**	−.278**	0.035	0.04	−0.1	.210**	−0.09	−.203**	−.897**	−			
15. Entrepreneur^**b**^	0.043	.110*	0.062	−0.062	.141**	.161**	−0.01	−0.039	0.06	0.042	−0.024	−0.031	−0.108	−.119*	−		
16. Unemployed^**b**^	0.094	0.062	−0.001	−0.034	−0.049	−0.003	−0.025	−0.067	.114*	.118*	−.116*	0.01	−.184**	−.202**	−0.024	−	
17. Income^a^	−0.03	−0.02	0.042	0.016	.504**	.253**	−0.05	0.038	0.021	−.246**	.107*	.236**	.713**	−.648**	0.082	−.215**	−

*Note.* ^*^p < .05, ^**^p < .01

^a^Pearson

^b^Spearman

^c^reference class women

### Moderating and mediating results

#### Connections of eco-anxiety with pro-environmental behavior.

First, we examined the role of background variables in the regression model explaining PEB (Table 3). Gender explained the amount of PEB statistically significantly (p < .001), with women reporting more PEB than men. Those living in a village reported less PEB than those living in a city (p = .027). Those living in a rural area reported less PEB than those living in a city (p = .045), but the difference was not statistically significant after adding eco-anxiety to the model (p = .088). Those with a doctoral degree reported more PEB than those with a university degree (p = .023). No statistically significant difference was found between those with comprehensive or secondary education and those with a university degree in the level of PEB (p = .396). Overall, background variables explained 8.9% of the variation in PEB when measured by the adjusted coefficient of determination. When eco-anxiety was added to the regression model, the explanatory power of the model increased by 10.6%. Eco-anxiety explained the level of PEB (p < .001): those who experienced more eco-anxiety performed more PEB. Together with the background variables, eco-anxiety explained 19.5% of the variation in the level of PEB, measured by the adjusted coefficient of determination. The results of our regression analysis also confirm our hypothesis that the level of eco-anxiety is connected with the levels of PEB (H1).

**Table 3:** Connections of eco-anxiety with PEB.

Effect	Estimate	*SE*	aR^2^	∆R^2^	*p*
Step 1			.089	.102^***^	
Gender^a^	−.56	.12			<.001
Rural^b^	−.35	.17			.042
Village^b^	−.45	.20			.027
Comprehensive or secondary education^c^	−.10	.12			.396
Doctoral studies ^c^	.49	.21			.023
Step 2					
Gender^a^	−.31	.12			.011
Rural^b^	−.28	.16			.088
Village^b^	−.38	.19			.047
Comprehensive or secondary education^c^	−.12	.11			.284
Doctoral studies^c^	.55	.20	.195	.106^***^	.006
Eco-anxiety	.34	.05			<.001

*Note.* ^*^p < .05, ^**^p < .01, ^***^p < .001

All continuous variables have been standardised. Unstandardised estimates are reported.

aR^2^ = adjusted coefficient of determination, ∆R^2^ = coefficient of determination change

^a^reference class women

^b^reference class urban environment

^c^reference class university degree

#### Moderating effect of social support on the association between eco-anxiety and PEB.

We conducted a second regression model to examine the possible moderating role of social support on the association between eco-anxiety and PEB (Table 4). Adding social support in the second step of the model increased the explanatory power of the model by 6.9%, the whole model explaining 15.7% of the variance in PEB. Social support explained the amount of PEB significantly (p < .001); those who received social support reported more PEB. Adding eco-anxiety to the model in the third step of the regression model increased the explanatory power of the model by 7.9 percentage points, explaining 23.5% of the variance in PEB. Eco-anxiety was a highly statistically significant (p < .001) explanatory variable of the variance of PEB in this second model, as well. In the fourth step of the regression model, an interaction between eco-anxiety and social support was added to the model to examine the moderating effect. The interaction was not found to explain the variance of PEB (p = .769), and adding the interaction to the model weakened its adjusted coefficient of determination by 0.2 percentage points (from 23.5% to 23.3%). The results indicate that social support was a moderately strong predictor of the variance in PEB, but it did not moderate the connection between eco-anxiety and PEB.

**Table 4:** Moderating effect of social support on the association between eco-anxiety and PEB.

Effect	Estimate	*SE*	aR^2^	∆R^2^	*p*
Step 1			.089	.102^***^	
Gender^a^	−.56	.12			<.001
Rural^b^	−.35	.17			.042
Village^b^	−.45	.20			.027
Comprehensive or secondary education^c^	−.10	.12			.396
Doctoral studies^c^	.49	.21			.023
Step 2			.157	.069^***^	
Gender^a^	−.46	.12			<.001
Rural^b^	−.28	.17			.092
Village^b^	−.43	.19			.027
Comprehensive or secondary education^c^	−.03	.12			.808
Doctoral studies^c^	.40	.21			.051
Social support	.67	.12			<.001
Step 3			.235	.079^***^	
Gender^a^	−.27	.12			.027
Rural^b^	−.23	.16			.146
Village^b^	−.38	.19			.043
Comprehensive or secondary education^c^	−.06	.11			.585
Doctoral studies^c^	.48	.20			.016
Social support	.53	.12			<.001
Eco-anxiety	.30	.05			<.001
Step 4			.233	.000	
Gender^a^	−.27	.12			.027
Rural^b^	−.23	.16			.147
Village^b^	−.37	.19			.045
Comprehensive or secondary education^c^	−.06	.11			.605
Doctoral studies^c^	.48	.20			.016
Social support	.52	.12			<.001
Eco-anxiety	.33	.09			<.001
Eco-anxiety x Social support	−.03	.11			.769

*Note.* ^*^p < .05, ^**^p < .01, ^***^p < .001

All continuous variables have been standardised. Unstandardised estimates are reported.

aR^2^ = adjusted coefficient of determination, ∆R^2^ = coefficient of determination change

^a^reference class women

^b^reference class urban environment

^c^reference class university degree

#### The mediating effect of social support on the connection between eco-anxiety and PEB.

**Fig 2:**
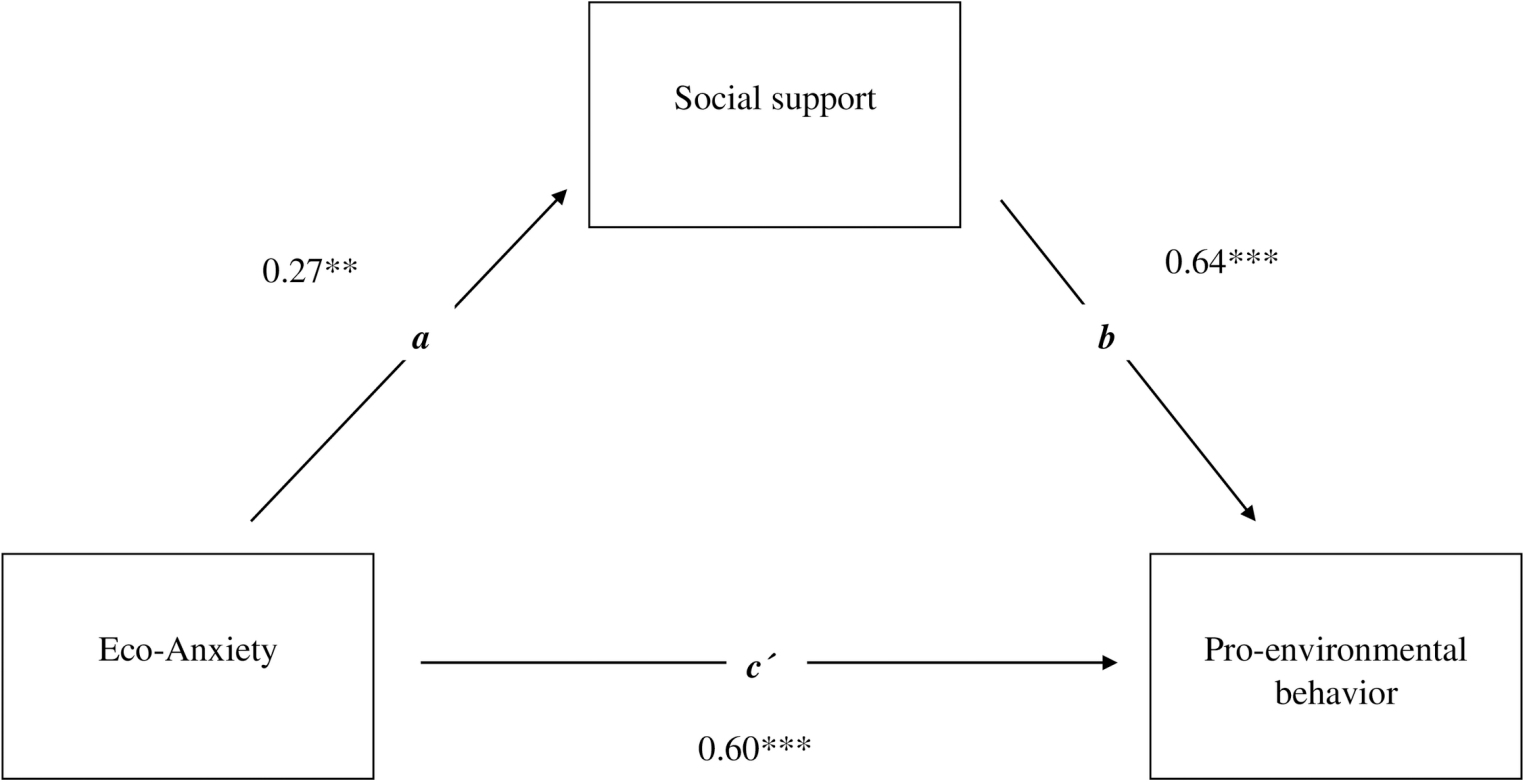
Mediation model that describes the mediating effect of social support on the relationship between eco-anxiety and PEB. ***p < .001 **p < .01; ab = .175; Confidence interval [.06;.36]; C = c´ + ab = .78***; n = 358.

The mediator model is based on the idea that the mediator does not moderate the relationship between x and y. Our results supported this assumption, allowing us to examine the mediating effect. We examined the mediating effect of social support using a simple mediation model (Fig 2). The background variables included in the analysis were gender, education level, and place of residence. Eco-anxiety had a statistically significant positive direct connection with the level of PEB (path c’ = 0.60, p < .001). Eco-anxiety was connected with seeking social support (path a = 0.27, p < .01), with those experiencing more eco-anxiety receiving more social support. Social support was found to be positively related to the level of PEB (path b = 0.64, p < .001). That is, those who received more social support performed more PEB. Social support also had a statistically significant mediating effect on the connection between eco-anxiety and PEB (path ab = .175) measured by a 95% bias corrected bootstrap confidence interval after controlling for background variables, with a non-zero confidence interval [.06;.36]. Since the connection was positive, the results suggest that social support mediated the effect of eco-anxiety on PEB. Thus, among those experiencing eco-anxiety, those who had social support were the most likely to perform PEB. The overall connection of eco-anxiety on PEB was statistically significant (path C = c´ + ab = .78, p < .001).

## Discussion

Environmental issues and the emotions they evoke are an increasingly topical challenge to human well-being. Our current study provides new insight into the use of social support – a coping strategy that has gotten limited attention in the increasing research on coping with eco-anxiety – and its associations with eco-anxiety and PEB. Our results demonstrate that experiencing eco-anxiety was associated with higher self-reported PEB. The use of social support as a coping strategy was associated with higher PEB. In addition, social support mediated the association between eco-anxiety and PEB. The participants who perceived social support available for their high levels of eco-anxiety reported more PEB. Our results suggest that by facilitating seeking and receiving social support we could both support coping with eco-anxiety and promote pro-environmental behavior.

### Eco-anxiety and social support associate with pro-environmental behavior

Higher levels of eco-anxiety were associated with a higher level of PEB. Eco-anxiety together with background variables explained about 20% of the variation of PEB. In addition to eco-anxiety, we found that social support as a coping strategy was associated with higher levels of PEB. Furthermore, social support was found to mediate the association between eco-anxiety and PEB, but no moderating role was found. The participants who experienced more eco-anxiety and perceived social support available were more likely to engage in PEB. The results suggest that receiving social support is an effective coping strategy for environmental emotions, especially for those who experience high levels of eco-anxiety. Receiving social support may release an individual’s resources to engage in PEB.

### Social support as a coping strategy to eco-anxiety

Contrary to our hypothesis our findings suggest that social support was associated with higher levels of eco-anxiety. This could be explained by the fact that those experiencing more eco-anxiety may need more social support and therefore seek it. It is also worth noting that those who did not receive social support and those who did not want or feel the need to seek social support were combined in the same category. Those experiencing less eco-anxiety may not need social support to cope with the emotions provoked by environmental issues. Considering our findings regarding the mediation, we propose that an individual experiencing eco-anxiety is more likely to seek support for environmental emotions from their social network. Perceived availability of social support may subsequently protect the well-being of an individual and thus may promote their engagement with PEB.

Earlier research has suggested that social support is divided into problem-focused and emotion-focused support [24] and that this division applies also to optimal coping strategies in the context of environmental issues [12]. Social support has been found to protect an individual from harmful stress [29] and to be associated with improved psychological well-being [25]. Social support has also been found to aim at corrective action [30] and is associated with PEB [25]. Social support seems to provide both solutions and means of action, as well as opportunities to cope with emotions and it protects the individual from harmful stress. Hence, social support could be an optimal coping strategy from the perspective of both the well-being of the individual and our planet. In addition to supporting the well-being of the environment, PEB itself can be seen as a problem-focused coping strategy [30]. As problem-focused coping has been thought to protect from the adverse effects of stress [30], it may support individual well-being as well, as proved by Bradley and colleagues [25]. It can therefore be argued that engaging in PEB is also an important coping strategy in the context of eco-anxiety; indeed, practicing pro-environmental behavior has been considered the most effective coping strategy (80%), with spending time in nature as the second (75%) and discussing the topic with others as the third (58%) most effective coping strategy, in a study by Hyry [35].

### Limitations and future research

A major limitation of this research is the psychometric properties of the measures. First, the social support variable is a dichotomous single-item measure. This measure does not reach the frequency of social support or if the participants feel supported by the network. It may be that an individual has access to social support but still does not turn to this support network when experiencing difficult environmental feelings. Our variable does not capture this aspect since it only measures the perceived availability of social support. Even though perceived social support alone has been considered beneficial [26,27] we need future research to cover both perceived and received social support in the context of environmental emotions. The social support variable was left-skewed, indicating that most participants reported access to social support. In addition, those who did not receive social support and those who did not want it were coded in the same category. These issues may complicate the interpretation of the results. The social support variable was constructed for this study and not based on predefined reliable variables. In future research, measuring social support with a variable that captures aspects neglected in our study would be valuable. Second, the eco-anxiety variable was likewise constructed for this study. Even though the reliability of our variable is considered good and is based on previous theories of eco-anxiety [7,14,15], using a variable developed in prior research would have improved the replicability of our study. Furthermore, the reliability of the PEB was poor. However, earlier study including 32 countries showed variation in Cronbach’s alphas across countries and samples (α range = 0.56–0.85) [1]. Thus, it is possible that poor reliability in our study is coincidence in our sample. In addition, poor reliability may stem from heterogeneity of different PEB domains, as stated earlier. Finally, using a previously validated Likert scale to capture the frequency of reported pro-environmental actions would have improved the psychometric properties of the measure of PEB.

Even though we measured social support with the question about having someone to discuss feelings related to environmental problems, our study design does not provide information on the form of the social support and whether the participant perceives the amount and quality of received social support as adequate. The received support can be emotion-focused, such as empathy and comfort, or problem-focused, such as information, advice, or concrete help to solve the problem. Further analysis is needed to examine the variety of supportive behaviors and their effects on psychological well-being and engagement in PEB.

Since our study is based on self-reported behavior, the responses may be influenced by social acceptability and memory biases. However, a meta-analysis by Kormos and Gifford [36] indicated that self-report measures of PEB have strong validity, although caution must be used in interpretation. As we used a voluntary online survey, it is likely that participants interested in the research topic were over-represented, limiting the results to environmentally aware adults.

Since we used a cross-sectional design, conclusions about causality between variables cannot be made. It is possible that, following the use of coping strategies, the emotions that individuals experience as a result of environmental issues change and thus either promote or discourage PEB. While this study contributes to the current knowledge on relationship between eco-anxiety and PEB and the importance of coping strategies – namely, social support – in this context, we highlight the need for longitudinal studies on the subject. In addition, this study used only quantitative methods. A mixed-method approach with qualitative and quantitative data could, for example, examine differences between environmental actions [31]. Furthermore, we only examined eco-anxiety, which includes several negative environmental emotions. Examining the relationship between PEB and positive environmental emotions and well-being could provide a broader understanding of how PEB affects an individual’s emotional well-being.

Our sample consisted of environmentally aware adults. However, the higher explanatory power of our study cannot be explained by mere knowledge, since it is not sufficient in motivating to adopt PEB [37]. Instead, environmental emotions have been shown to mediate the connection between environmental knowledge and action [37]. Thus, the interaction between environmental awareness and environmental emotions could explain our findings on the association between eco-anxiety and PEB. It would be important to examine these research questions with a sample of participants with lower levels of environmental knowledge.

In our data, people with higher education, women, and those living in an urban environment were over-represented. Specifically, unequal gender distribution in our sample (i.e., with 76.4 being women) could have influenced our results, considering that previous studies have shown gender differences in eco-anxiety and PEB, with women reporting both more frequently than men [1,17,22]. It would be important to repeat the study with a more representative sample for a more comprehensive picture of how eco-anxiety, PEB, and social support manifest themselves across the population. In addition, our study is only generalizable to environmentally aware Finnish adults. So far, in Finland, the effects of environmental problems, such as climate change, have mostly been indirect (i.e., exposure to the climate crisis in the media), unlike in Global South, for example, where the impacts have been more direct and severe (e.g., floods, drought). Since Finnish adults are more likely to be exposed to the indirect effects of environmental problems, the optimal coping mechanisms may differ from coping strategies used by people exposed to the direct effects. Moreover, social support is a concept that is strongly influenced by culture, and thus our findings cannot be generalized cross-culturally.

It is often argued that pro-environmental behavior reduces eco-anxiety. However, it is crucial to investigate other coping strategies as well, since coping focused on emotion regulation is important to prevent burnout in people constantly striving to do more for the environment [6]. The increasing visibility and awareness of environmental issues and their effects are likely to increase the number of individuals experiencing eco-anxiety. Knowledge of appropriate coping strategies for dealing with environmental emotions may therefore enable the development of interventions to support coping strategies and reduce eco-anxiety. Based on the results of this study, interventions that provide access to social support for dealing with environmental emotions could be particularly useful for reducing eco-anxiety.

## Conclusions

Our study is, to the best of our knowledge, among the first ones to explore the significance of using social support to cope with eco-anxiety, and the connections between social support, eco-anxiety, and pro-environmental behavior (PEB). As such, our study contributes to the increasing body of research focusing on coping in the context of environmental problems. The results of this study indicate that the participants who experienced eco-anxiety to a higher degree and had social support available were more likely to report PEB. Our results suggest that social support is an effective coping strategy for both regulating environmental emotions and promoting sustainable behavior, especially when it includes both solution-focused and emotion-focused support. These findings can provide valuable insights for designing interventions aimed both at increasing behavioral engagement in the context of pro-environmental behavior and at supporting constructive coping with emotions related to environmental problems. We argue that by promoting the processing of environmental emotions and the use of prosocial coping strategies, the well-being of the individuals as well as the well-being of the environment is enhanced.

## References

[1] Ogunbode CA, Doran R, Hanss D, Ojala M, Salmela-Aro K, van den Broek KL. Climate anxiety, wellbeing and pro-environmental action: correlates of negative emotional responses to climate change in 32 countries. J Environ Psychol. 2022;84:101887.

[2] Hogg TL, Stanley SK, O’Brien LV, Wilson MS, Watsford CR. The hogg eco-anxiety scale: development and validation of a multidimensional scale. Global Environ Change. 2021;71:102391.

[3] Clayton S. Climate anxiety: psychological responses to climate change. J Anxiety Disord. 2020;74:102263. doi: 10.1016/j.janxdis.2020.102263 32623280

[4] Cianconi P, Betrò S, Janiri L. The impact of climate change on mental health: a systematic descriptive review. Front Psychiatry. 2020;11(74):1–15. 32210846 10.3389/fpsyt.2020.00074 PMC7068211

[5] Martin G, Reilly K, Everitt H, Gilliland JA. Review: the impact of climate change awareness on children’s mental well‐being and negative emotions – a scoping review. Child and Adolescent Mental Health. 2021;27(1). 10.1111/camh.12525 34873823

[6] Pihkala P. Johdatus ympäristöahdistukseen: ympäristöongelmien psyykkiset vaikutukset. Tieteessä tapahtuu. 2018;36(6):31–8.

[7] Fritze JG, Blashki GA, Burke S, Wiseman J. Hope, despair and transformation: Climate change and the promotion of mental health and wellbeing. Int J Ment Health Syst. 2008;2(1):13. doi: 10.1186/1752-4458-2-13 18799005 PMC2556310

[8] Marks E, Hickman C, Pihkala P, Clayton S, Lewandowski ER, Mayall EE. Young people’s voices on climate anxiety, government betrayal and moral injury: a global phenomenon. SSRN Electronic J. 2021. 10.1016/S2542-5196(21)00278-3 34895496

[9] Heeren A, Mouguiama-Daouda C, Contreras A. On climate anxiety and the threat it may pose to daily life functioning and adaptation: a study among European and African French-speaking participants. Climatic Change. 2022;173(1–2). 10.1007/s10584-022-03402-2 PMC9326410 35912274

[10] Stanley SK, Hogg TL, Leviston Z, Walker I. From anger to action: Differential impacts of eco-anxiety, eco-depression, and eco-anger on climate action and wellbeing. J Climate Change and Health. 2021;1(1):100003.

[11] Clayton S, Karazsia BT. Development and validation of a measure of climate change anxiety. J Environl Psychology. 2020;69(101434):101434. https://www.sciencedirect.com/science/article/abs/pii/S0272494419307145?via%3Dihub

[12] Doherty TJ, Clayton S. The psychological impacts of global climate change. Am Psychol. 2011;66(4):265–76. doi: 10.1037/a0023141 21553952

[13] Hickman C. We need to (find a way to) talk about … Eco-anxiety. J Social Work Practice. 2020;34(4):411–24. doi: 10.1080/02650533.2020.1844166

[14] Ojala M, Cunsolo A, Ogunbode CA, Middleton J. Anxiety, worry, and grief in a time of environmental and climate crisis: a narrative review. Ann Rev Environ Res. 2021;46(1):35–58.

[15] Kurth C, Pihkala P. Eco-anxiety: What it is and why it matters. Front Psychol. 2022;13:981814. doi: 10.3389/fpsyg.2022.981814 36211934 PMC9537110

[16] Baudon P, Jachens L. A scoping review of interventions for the treatment of eco-anxiety. Int J Environ Res Public Health. 2021;18(18):9636. doi: 10.3390/ijerph18189636 34574564 PMC8464837

[17] Anderson DJ, Krettenauer T. Connectedness to nature and pro-environmental behaviour from early adolescence to adulthood: A comparison of urban and rural Canada. Sustainability. 2021;13(7):3655. doi: 10.3390/su13073655

[18] Whitmarsh L, Poortinga W, Capstick S. Behaviour change to address climate change. Curr Opin Psychol. 2021;42:76–81. doi: 10.1016/j.copsyc.2021.04.002 33991862

[19] Mesmer-Magnus J, Viswesvaran C, Wiernik BM. The role of commitment in bridging the gap between organizational sustainability and environmental sustainability. In: Jackson SE, Ones DS, Dilchert S, editors. Managing human resources for environmental sustainability. Jossey-Bass/Wiley. 2012: 155–86.

[20] Ones DS, Wiernik BM, Dilchert S, Klein R. Pro-Environmental Behavior. Int Encyclopedia of the Social & Behav Sci. 2015;82–8. https://www.sciencedirect.com/science/article/pii/B9780080970868220084

[21] Sangervo J, Jylhä KM, Pihkala P. Climate anxiety: Conceptual considerations, and connections with climate hope and action. Global Environ Change. 2022;76:102569.

[22] Roeser S. Risk communication, public engagement, and climate change: a role for emotions. Risk Analysis. 2012;32(6):1033–40. 22519693 10.1111/j.1539-6924.2012.01812.x

[23] Lazarus RS, Folkman S. Stress, appraisal, and coping. New York: Springer Publishing Company. 1984.

[24] Carver CS, Scheier MF, Weintraub JK. Assessing coping strategies: a theoretically based approach. J Pers Soc Psychol. 1989;56(2):267–83. doi: 10.1037//0022-3514.56.2.267 2926629

[25] Bradley G, Reser J, Glendon I, Ellul M. Distress and coping in response to climate change. In: Kaniasty K, Moore KA, Howard S, Buchwald P, editors. Stress and anxiety: applications to social and environmental threats, psychological well-being, occupational challenges, and developmental psychology. Berlin: Logos Verlag Berlin. 2014.

[26] Eagle DE, Hybels CF, Proeschold-Bell RJ. Perceived social support, received social support, and depression among clergy. JSocial and Personal Relationships. 2018;36(7):026540751877613.

[27] Wethington E, Kessler RC. Perceived support, received support, and adjustment to stressful life events. J Health Soc Behav. 1986;27(1):78–89. 3711634

[28] Dumont M, Provost MA. Resilience in adolescents: protective role of social support, coping strategies, self-esteem, and social activities on experience of stress and depression. J Youth and Adolescence. 1999;28(3):343–63.

[29] Barrera M. Distinctions between social support concepts, measures, and models. Am J Community Psychology. 1986;14(4):413–45.

[30] Reser JP, Bradley G, Ellul M. Coping with climate change: Bringing psychological adaptation in from the cold. In: Molinelli B, Grimaldo V, editors. Handbook of the Psychology of Coping. Nova Science Pub Incorporated. 2012.

[31] Author. Details withheld to preserve blind review. 2019.

[32] Rees JH, Klug S, Bamberg S. Guilty conscience: motivating pro-environmental behavior by inducing negative moral emotions. Climatic Change. 2015;130(3):439–52. https://link.springer.com/article/10.1007/s10584-014-1278-x

[33] Nunnally JC. Psychometric theory. 2d ed. New York: McGraw-Hill; 1978.

[34] Preacher KJ, Hayes AF. Asymptotic and resampling strategies for assessing and comparing indirect effects in multiple mediator models. Behavior Research Methods. 2008;40(3):879–91. 18697684 10.3758/brm.40.3.879

[35] Hyry J. Kansalaiskysely ilmastonmuutoksen herättämistä tunteista ja niiden vaikutuksista kestäviin elämäntapoihin Kantar TNS Oy. 2019. Available from: https://www.sitra.fi/app/uploads/2019/08/ilmastotunteet-2019-kyselytutkimuksen-tulokset.pdf

[36] Kormos C, Gifford R. The validity of self-report measures of proenvironmental behavior: a meta-analytic review. J Environ Psychol. 2014;40(1):359–71.

[37] Carmi N, Arnon S, Orion N. Transforming environmental knowledge into behavior: the mediating role of environmental emotions. J Environ Educ. 2015;46(3):183–201.

